# How do parents experience being asked to enter a child in a randomised controlled trial?

**DOI:** 10.1186/1472-6939-10-1

**Published:** 2009-02-16

**Authors:** Valerie Shilling, Bridget Young

**Affiliations:** 1 Division of Clinical Psychology, School of Population, Community and Behavioural Sciences, Whelan Building, Quadrangle, University of Liverpool, Brownlow Hill, Liverpool, L69 3GB, UK

## Abstract

**Background:**

As the number of randomised controlled trials of medicines for children increases, it becomes progressively more important to understand the experiences of parents who are asked to enrol their child in a trial. This paper presents a narrative review of research evidence on parents' experiences of trial recruitment focussing on qualitative research, which allows them to articulate their views in their own words.

**Discussion:**

Parents want to do their best for their children, and socially and legally their role is to care for and protect them yet the complexities of the medical and research context can challenge their fulfilment of this role. Parents are simultaneously responsible for their child and cherish this role yet they are dependent on others when their child becomes sick. They are keen to exercise responsibility for deciding to enter a child in a trial yet can be fearful of making the 'wrong' decision. They make judgements about the threat of the child's condition as well as the risks of the trial yet their interpretations often differ from those of medical and research experts. Individual parents will experience these and other complexities to a greater or lesser degree depending on their personal experiences and values, the medical situation of their child and the nature of the trial. Interactions at the time of trial recruitment offer scope for negotiating these complexities if practitioners have the flexibility to tailor discussions to the needs and situation of individual parents. In this way, parents may be helped to retain a sense that they have acted as good parents to their child whatever decision they make.

**Summary:**

Discussing randomised controlled trials and gaining and providing informed consent is challenging. The unique position of parents in giving proxy consent for their child adds to this challenge. Recognition of the complexities parents face in making decisions about trials suggests lines for future research on the conduct of trials, and ultimately, may help improve the experience of trial recruitment for all parties.

## Background

When parents are asked to consider enrolling their child in a paediatric clinical trial their primary responsibility is the best interests of their child. This role is very different to an adult deciding about their own participation in a trial, whose role involves exercising individual autonomy [1]. Grappling with complex, medical information is always part of trial decision making but when this is on behalf of a vulnerable and dependent child, rather than to serve one's own rights and interests, the decision will likely feel more serious and possibly overwhelming. Despite this fundamental difference between trials in adult and paediatric medicine, relatively little is known about how the particular role of parents influences the communication about a trial. Insight into this is important if we are to improve families' experiences of trial recruitment, as well as optimise enrolment procedures and suggest appropriate directions for future research.

In this paper we will argue that when parents are approached about a trial for their child, the decision is influenced by particular considerations and requires special management. We will discuss evidence on the complex experience of parents when considering trial entry for their child, what trials mean to them and what contextual factors impact on their experience of decision making. We have drawn primarily on studies which have a qualitative design, though we have included a wider body of evidence where it offered useful insights. Studies using qualitative methodology were specifically targeted because they access participants' accounts in their own words, allowing more scope to explore their meanings and experiences and making fewer assumptions about what is important to them [2].

This discussion does not aim to make specific recommendations for how to discuss randomised clinical trials with parents, rather its intention is to increase awareness of how parents make sense of trials and suggest directions for future research to improve the experience of recruitment to trials from the perspective of all parties involved.

## Discussion

### Vulnerability, responsibility and regret

For parents the diagnosis of serious illness in a child can be a shattering experience [3]. When consent is sought soon after diagnosis, parents will be making decisions when they are distressed and vulnerable [4-6], whilst simultaneously trying to comfort their sick child. Being approached about a trial confronts parents with a volume and complexity of information well beyond their everyday experience and they can never be certain about what is the 'right' decision. However, whilst they may be vulnerable, protecting their child is fundamental to the parental role and this will shape how they think about trials. Parents in one study felt personally and directly accountable for their child's outcome on a trial and thought that giving consent for a child would be much more difficult than deciding to take part in a trial themselves [7]. Survey evidence confirms that the responsibility to act in the best interests of one's child is keenly felt, with one third of parents in one study reporting that while they might accept certain research risks for themselves they were much less certain about accepting the same risks for their baby [8].

The sense of responsibility that accompanies parents' decision making about trials may paradoxically render them more vulnerable, especially to the anticipation of regret [9]. Mothers whose children were enrolled into a bone marrow transplant trial dreaded the possibility that they might have to live with the knowledge that they had made the 'wrong' decision and this was intensified when things did not go well for the child [10]. Mothers of children with leukaemia [11] felt highly dependent on clinicians and experienced considerable unease in making decisions about trials with little grasp of the disease. People are motivated to avoid regret by making decisions that minimise its likelihood [12] and the more important the outcome, the greater the anticipation of regret particularly if the outcome is irreversible [13]. In view of the roles and responsibilities of parenthood, the anticipation of regret about potentially 'failing to protect' one's child may be a major influence on parents' decisions about trials. Canvin and colleagues speculate that the sense of responsibility surrounding the trial decisions of parents of children with epilepsy was eased by the voluntary and reversible nature of the decision [14]. However, when things do go wrong, people experience more regret for acts of commission (doing) than for acts of omission (not doing) [15], and think more about harm caused by direct action than about indirect harm [9]. Therefore, parents may hesitate to vaccinate their child even under conditions where the likelihood of a fatal side effect is only a fraction of the death rate from the disease [16], presumably because anticipated regret looms larger for the act of vaccination. As yet no research has looked at whether parents respond in the same way to trial participation – rationalising that not participating minimises the chance of regret and is the 'safest' option.

Understandings of events can, of course, be retrospectively shaped and reconstructed to manage regret. Parents who consented their child to enter the ECMO trial [17] constructed randomisation as offering protection from responsibility should the child have a poor outcome. This protection could be extended to doctors, who in the eyes of some parents could not be held responsible when there was uncertainty as to the best treatment. Some families constructed their views about which is the 'best' arm of the trial based around which arm they were allocated to – for example, some who were allocated to conventional treatment focussed on the likelihood that this avoided the risks associated with the treatment arm and need for transfer to another unit [17].

Recognising this difficult situation for parents, the incompatibility of wanting to do what is best for the child but not knowing what the best course of action is, may be critical in understanding how parents respond to clinical trials. What may seem to the trialist as a misunderstanding of trial rationale may be the parent constructing the situation in a way which is acceptable to their need to protect their child.

### The parental role and the function of consent

Decisions about trial entry may be a source of intense emotional strain for parents but being offered options about their child's treatment and research participation is likely to serve important psychological functions, particularly at times when there is little else parents can do instrumentally for the child. Parents of children hospitalised for spinal surgery described the "loss of parental role" and the accompanying helplessness and frustration they experienced as being of a similar magnitude as their distress about the potential of poor surgical outcome and watching their child in pain [18]. The process of consent itself could function as a symbolic acknowledgement of their role when there is little else parents can do.

When asked their views on Zelen randomisation (single consent method), parents whose baby was involved in a conventionally randomised trial were evenly divided on its acceptability [19]. With this method, randomisation takes place prior to discussion with the family. If the child is allocated to the conventional treatment arm, they are not informed about the trial. Only if the child is allocated to the experimental arm is consent sought. In offering 'protection' from the difficulties of trial decision making, the knowledge that there are treatments which they cannot access, and from the knowledge that treatment is determined by chance, this approach might be considered 'kinder' to those families allocated to the conventional treatment arm. However, Zelen randomisation was viewed negatively by some parents because they perceived it as preventing the sense that they were operating in the best interests of their child. As difficult as the decision on trial entry was for parents, they felt it was theirs to make [19]. Similarly, parents who participated in a survey investigating different types of consent to hypothetical neonatal resuscitation trials were more comfortable with prospective consent than with consent which was deferred, waived or required the parent to 'opt-out'. They wanted to be informed about the trial and make the decision themselves [20]. Other surveys provide further evidence that parents value their role in decisions about trial entry – over 90% say they do not want the doctor or nurse to decide on their behalf [21,22,8]. Parents upheld these views even when they realised that the quality of their consent was limited or experienced the informed consent process as an additional burden [23], indicating that consent could indeed serve important symbolic functions. Research on trials which involve deferred or waived consent [24] could further inform ideas about the social function of consent and processes of regret, both of which might be configured rather differently depending on whether consent is conventional, waived or deferred.

Bioethicists and others have pointed out that informed consent did not develop to safeguard autonomy, rather its primary purpose is to preserve well-being [25,26]. Allmark and Spedding suggest that parental consent for clinical trials is important as a social recognition of the role of parents, but offers little additional protection for children to that provided by appropriate research ethics, safety monitoring and governance procedures. From this position the quality of parental consent might be less critical, and their decision making difficulties could be eased by knowing about ethics [23] and other research safety and governance procedures. Of course, this assumes parents can be confident in the protection offered by research governance and monitoring but a recent review suggests that safety monitoring in paediatric trials may have been inadequate in some cases and highlights the need for monitoring of all paediatric trials [27]. At the same time as wanting the option to decide, parents value doctors' advice about trials [28] and some look to share responsibility for their decisions with practitioners. In one neonatal study three-quarters of parents believed their doctors would not ask to do research if it might put babies in real danger [8] and half indicated that they trusted their doctor and if he or she suggested their baby should enter a trial, they would agree. Though they have authority for their child, parents lack expertise in matters related to the child's illness, and as a consequence, are likely to rely heavily upon the expertise of medical practitioners.

The role of parents in trial decision making is therefore complex – they can be simultaneously responsible for their child and dependent on practitioners. The challenge for trialists in discussing paediatric trials with parents is to balance the tensions this brings, so supporting parents' decision making appropriately and guarding against the possibility of regret [7]. Research on how this might be achieved is much needed. The potential for emotional conflict is apparent in the accounts of parents of babies receiving treatment on NICU [29], who were united in wanting to be considered the primary decision-maker for their baby, but were much less consistent in wishing to be actively involved in decisions which they viewed as serious. The authors suggest that asking parents to do so could carry high 'psychological costs'.

### Threat, hope and certainty

The seriousness of the child's condition and the urgency surrounding trial entry will be important influences on how parents experience recruitment to trials, their sense of vulnerability, and the success of communication [23]. In turn, these will have consequences for whether parents retain a sense that they have operated in their child's best interests [30]. Parents considering oncology and neonatal trials report particularly high levels of distress during trial discussions and a sense that this impairs their ability to ask questions or seek additional information [31] occasionally leading them to later doubt their decisions [32]. In these situations the thoughts and emotions of parents are dominated by the fact that they have a very sick child [33] or worse, the fear that their child might die [34]. By contrast, parents of children with less serious chronic illnesses report more comfort in making decisions about trial entry [32] and in judging what is in the best interests of the child because they have experience of the disease. It follows that parents' priorities will vary with the seriousness of their child's illness. For example, parents of children participating in research on mild to moderate asthma rated "learn more about disease" as the primary motivation (from 14 choices) [35]. In chronic childhood illness, the day to day management of the condition, the child's quality of life and the simple practicalities of trial participation may gain importance. This is in marked contrast to parents of children whose lives were in the balance, some of whom struggled even to understand or recall that research participation was voluntary [10].

Parents' worries and misperceptions provide further insights into what trials mean to them and point to specific sources of difficulty in communication. The worry that their child might be randomized to the less effective treatment [31] and the responsibility that they would feel if the child later deteriorated are particularly common [1]. On finding that their child has been allocated to the standard treatment arm, parents sometimes report a sense of disappointment or missed opportunity, as if the child has been deprived of a known beneficial treatment or has 'failed' the selection process. Some mothers felt resentment that they had been asked to go through the trauma of deciding about trial entry for 'nothing' [11]. Others viewed randomisation as a way of allocating limited resources and expressed guilt that their child had been allocated the experimental arm while others had only received conventional treatment [17,36]. Founded on a difficulty in accepting that there is uncertainty about the effectiveness of trial treatments or an assumption that the experimental treatment will necessarily be superior, these responses remind us that in the face of intense threat and uncertainty, parents look for hope and certainty. In such circumstances it is not surprising they find the uncertainty that is intrinsic to trials challenging both cognitively and emotionally, or that they are liable to construct trial treatments as the answer to their child's needs.

The generally high rates of recruitment to trials in neonatology [37] and childhood cancer [38] suggests the level of threat and parents' consequent needs for hope could be important influences on how they view trials. Parents of chronically and terminally ill children stated that they were prepared to take greater risks in treatment in the hope of a cure [7]. Similarly, parents' decisions about Phase 1 cancer trials, which can bring side-effects and offer little chance of long term medical benefit, may turn upon a fear of 'giving-up' on their child and the need to 'leave no stone unturned'[39,40]. High recruitment rates may also be testament to the importance that is deservedly placed on research in neonatology and childhood cancer by both parents and trial staff [41,42]. But they could also be a sign of parents' reluctance to decline a trial where it involves saying "no" to the clinical team on whom their child's care depends. By contrast parents of healthy children considering participation in vaccine research believed that children should only take part in research where the medical benefits outweigh any potential risk [43]. Work comparing what researchers say and intend when they discuss trials with parents, and what parents interpret from these discussions will yield further insights into the challenges for both parties [44] and how they co-construct their views of trials and their respective roles.

In deciding about hypothetical research scenarios, parents and children were more likely to respond intuitively and emotionally to research information than to systematically attempt to consider and weight all the available information. The result was that the 'information' participants used to underpin their decisions was often very different to the information that the researchers intended them to use [45]. Participants' emotional responses to information were shaped by their particular life experiences. Similarly, Chantler and colleagues report that parents who received the same information about a vaccine study varied widely in their perceptions of the risks of the trial, with some seeing it as potentially dangerous while others viewed it as safe with no negative effects. Parents from a professional background in science or ongoing experience of medical care were less likely to cite anxiety and unfamiliarity as a reason for declining the trial, and in the view of the authors, were more open to research and better able to judge the trial's importance and value [43].

If the same information can mean different things to different people, clinicians' assessment of risk will also be influenced by their professional experience, and trial risks as seen by parents may be very different to those perceived by trial staff. Moreover, relative risk, a concept familiar to clinicians accustomed to the uncertainty that is intrinsic to medical practice, could seem very different to parents for whom even improbable risks may be intolerable [46]. Parents and investigators agreed that the risks associated with participation in clinical anaesthesia or surgery research was the most important information to disclose, but parents were more convinced than the investigators of the importance of disclosing the benefits of the research [47]. One interpretation is that risks resonate more deeply with parents and they therefore need to balance these with positive information. Similarly, clinicians viewed the CANDA trial as a low risk, routine trial of broadly equivalent interventions [48] but many parents consenting to the trial viewed it as offering an otherwise unavailable option which might save their baby's life. To the clinician research can be considered low risk when it involves no greater risk than conventional treatment. To the parent everything is high risk because they have a sick child.

The contrast between parents' and clinicians' and trialists' views warrants further investigation but it is unlikely that parents always approach decisions about trials in the way imagined by some – a rational and mechanistic process of weighing up all of the available information before reaching a decision [45,49]. Associated with a 'rational choice' model of trial decision-making is the assumption that communication is a mechanistic and technical matter of assembling and transmitting the 'correct' information but successful communication about trials or any other topic is more than a technical matter [50]. It is a dynamic, social and emotional process in which the parties interact to construct meaning. Their interaction will be shaped by the particular context and nature of the trial, the hopes, fears, expectations and obligations the parties bring to the trial discussions, and those that continually emerge during the course of discussions and in their later reflections.

### Individual and group differences in trial perceptions and information preferences

Even in life threatening situations, not all parents will experience trials as adding "stress to an already stressful situation" [22]. Wide individual differences in parents' responses to being approached about trials have been reported with 20% reporting that the trial reduced their anxiety, 56% felt it made no difference and 24% reporting that the trial increased their anxiety [51]. Associations between anxiety and the decisions parents make about trials have also been observed. Parents who decline trials report higher anxiety, rate the risks of the trial as higher [52] and are more likely to fear randomisation [53], but one study found consenting parents were more anxious than those who declined [54]. Rather than being straightforward the relationship between anxiety and trial decisions may be mediated or moderated by factors such as trust in medical research [55] and the parent-practitioner relationship [56].

Parents also vary in their preferences for trial information [57], some wanting more information whilst others feel overwhelmed by information and want less [58]. Miller and colleagues reported a trend for more information to be associated with greater parental anxiety and less control [59], which may be exacerbated by detailed information on the toxic effects of treatment or mortality rates. Of course, the anxiety experienced by parents will itself impact on their experience and interpretation of the trial information and discussion [60]. In one study of parents of children considering an oncology trial, those with higher levels of anxiety were more likely to report that the risks associated with trial entry were not clearly explained [61]. Getting the level of information right is an important challenge – one study of parents' attitudes to their child's enrolment in a trial of antiretroviral therapy reported a significant relationship between perceiving that information was lacking and unwillingness to enrol in future trials [62]. Similarly Tait and colleagues report that parents who were more satisfied with the clarity and quantity of trial information were more positive about the trial than parents who were dissatisfied with the information [63]. Researchers have suggested a 'goodness of fit' approach between the person and the amount of information given [64].

Discussions about trial participation may have lasting effects for those who decline trials as well as those who accept. Non participants report inaccurate [65] or even harmful misconceptions [48] after being approached about research. Those conducting trials need to understand the processes that underpin these difficulties if they are to be ameliorated. Concern that those who decline or drop out may come to regret their decision [65] might be extended to consider how individuals approached about trials may differ in their tendency to trust others. Evidence suggests that a substantial minority of people find it difficult to trust other people [66] and are less likely to perceive relationships with health professionals as supportive [67]. Such people may be especially at risk of harmful misconceptions if trialists cannot vary their approach according to individual needs. Indeed, the assumption that 'good' communication is synonymous with providing information that is ever more specific or systematic runs counter to the requirement, in complex discussions about trials, to select and tailor according to individual need [59]. There is, of course, a baseline level of information necessary for informed consent [25]. Beyond this, trialists and ethicists could consider how far communication about trials can be configured around the needs and preferences of individual parents [68] as well as the social norms of the setting [25].

Of the few studies that have considered group differences in trial recruitment, most have focussed on families of children with leukaemia. How parents from different socio-demographic backgrounds *experience *trials has not been explored, though researchers have investigated group differences in rates of trial participation and researcher-defined understanding of trials. Miller and colleagues [59] report that low socio-economic status (SES) and membership of a minority ethnic group (in this case largely Hispanic) was associated with lower understanding of key components of informed consent. A related study reported no differences in likelihood to enrol a child in a trial between English speaking majority parents, English speaking minority parents and non-English speaking minority parents [69]. Parents in the non-English speaking group asked fewer questions than parents in the other groups. The authors link this to Latino cultural norms such as 'respeto' and 'fatalismo' which discourage questions and interactivity and encourage the idea that God will decide the fate of the child [70]. Emphasising the diversity that also exists *within *different ethnic groups [70] the authors point out that such traditional values are far from uniform within Hispanic groups and tend to be less common in immigrant groups with high SES.

Beyond oncology, research on group differences is limited. Harth and Thong reported that, among other characteristics, parents who put their child forward for an asthma trial were less educated and from less advantaged backgrounds than parents who declined [71] while a study of factors influencing consent to a neonatal trial found no such differences [28]. Tait and colleagues [72] report that greater understanding of 11 key elements of consent (to clinical anaesthesia or surgery trials) was positively associated with parents' educational level but not ethnicity and that understanding was higher in consenters than non-consenters.

Of course, a parent can only understand and recall information if it has been provided in the first place. Miller and colleagues [59] report that practitioners provided less information to ethnic minority parents and parents with low SES and were less likely to ask their opinion or encourage questions. Simon and colleagues [69] also found that non English speaking minority groups were given less information and clarification of features such as randomisation and right to withdraw than either the English speaking majority or minority groups.

Noting how the content of trial discussions and researcher-defined quality of the consent were linked to parental ethnicity or clinician attitude towards parental ethnicity, Simon and Kodish [70] emphasise the danger of making assumptions based on ethnicity or socio-economic factors, which may contribute to the omission of important information for families. However, reports that lower SES is related to less information giving by practitioners, but also to lower anxiety and a greater sense of control among parents [59] point to competing interpretations of group variations. They could represent clinicians' attempts to empower parents by tailoring communication to their needs and avoiding overwhelming them with information.

While it is often reported that parents from minority groups or disadvantaged backgrounds are less likely to understand trials (e.g. [73-75]), little research has investigated how parents from different social and ethnic groups *experience *being asked to consider a trial for their child. Research of this sort could inform efforts to meet the needs of families from different backgrounds when they are approached about trials.

Although qualitative research on the attitudes, understanding and concerns about medical research has been conducted amongst adults in developing countries (e.g. [76]), the authors are not aware of any qualitative studies that report the *experience *of being asked to consider a randomised controlled trial for a child, from the perspective of parents in developing countries. Important questions surround the recruitment of children to trials in developing countries and researchers conducting such trials undoubtedly face particular challenges in managing recruitment in societies which are more family and community orientated and less individualistic than developed countries [76]. This represents another important gap in the literature.

### Faith and trust in medical care

Good relationships and communication between parents and their clinician offer parents a sense of understanding, safety and trust [59] which in turn may influence decisions about trial entry [77]. Parents considering a cardiac trial expressed a preference for the trial to be explained to them by their cardiologist or surgeon rather than the principal investigator or the research coordinator suggesting that the clinical relationship provides some comfort for families when considering trials [78]. By contrast, lack of confidence in the doctor has been associated with increased difficulty in decision making about trial entry [1]. The relationship with the clinician and its impact on the decision making process may be different across trials. Whilst oncology and neonatology tend to see good rates of trial participation, trial discussions in these settings often take place within hours or days of the family's first meeting with the clinical team suggesting that trust is not necessarily dependent on the longevity of a relationship. Indeed, community-based trials where families will likely have long-standing relationships with their doctors tend to see much lower rates of participation [37]. Evidence that doctors who have long-standing relationships with families fear that an approach about research might damage the relationship [79] and might therefore be inclined to present trials less favourably, throws some light on these findings though more research is needed comparing trials conducted in different clinical settings to better interpret these patterns.

Others suggest that timing is crucial and discussing research may seem irrelevant and even insensitive in the immediate aftermath of diagnosis of life-threatening illness, unless handled in a way that allows families to feel the overall care and wellbeing of the child is the clinician's main aim [80]. But this raises yet another tension for trialists to negotiate – the therapeutic misconception. Thought to be at the centre of one of the most resilient misunderstandings about research, the therapeutic misconception describes the belief that the purpose of a clinical trial is to benefit the individual patient rather than gather data to develop scientific knowledge (National Bioethics Advisory Commission referenced in [81]). It can be a considerable struggle for patients and families to separate discussions about treatment options and management from those about trial participation [82,4,10], particularly when faced with serious illness. Adult patients with advanced cancer interpreted their doctor's offer to participate in a Phase 1 trial as meaning that the trial was the right thing for them [83]. However, the therapeutic misconception may itself be a mislabelling of the situation: a parent's hope that a child may receive a treatment that is later shown to be better than the standard treatment is not fundamentally misinformed, as this is itself the purpose of the trial [23]. The other features of trials such as follow-up visits and extra time spent discussing the child's condition are often perceived by parents as direct benefits of participation (e.g. [84-87]). Given the complexities of trial communication when a child's life is threatened, the therapeutic misconception does not necessarily reflect a straightforward failure of the researcher to adequately explain the research methodology or of the parent to understand it. Indeed, it may be harmful for parents to abandon the therapeutic misconception altogether, as doing so could be tantamount to losing hope and trust in medical practitioners. As Snowdon and colleagues suggest, where care and research become completely disconnected in people's minds, the result may be an "injurious misconception" whereby simply being approached about a trial triggers a disproportionate sense of risk and threat [48].

### Putting others first?

Altruism is frequently cited by researchers and parents as a motivation for consenting to paediatric trials, but the weight of the obligation to protect one's child means it is unlikely that it is an overwhelming consideration when parents are deliberating whether to enter their child in a trial. Regardless of whether they declined or accepted, the primary concern for parents considering an anaesthesiology trial was the child's safety [88]. Similarly, while parents believe that clinical research with children is important, they do not necessarily wish to involve their own children in that research [7] and their primary objective is to protect their child from harm [8]. When considering hypothetical, non-beneficial research, Reynolds and Nelson report that parents and children would quickly evaluate the procedural risks of a study and reject or provisionally accept on that basis before considering reasons for participation such as altruism [45]. Hence, fathers of children with cancer were far more likely to reinforce altruism as a reason for research participation when the research was non-invasive but where the research involved the child physically, altruism was not seen as a sufficient reason for taking part [5]. Parents of children in Phase 1 cancer trials where the benefits to others most clearly outweigh the individual gains, spoke of 'carrying on the fight' and 'not being ready to let go', while others clung to the hope of a miracle cure or buying enough time for some other treatment to become available [89]. Understandably, participating in such a trial was seen by parents as a way to do everything they could for their child.

Nevertheless, around two thirds of discussions between parents and practitioners about childhood cancer trials contained some mention of altruism, though it was usually raised by clinicians rather than families [90]. There was no evidence that the mention of altruism affected trial recruitment rates suggesting that it was not coercive, but a number of other studies report altruism to be a motivating factor for parents considering trial entry [28,91,92]. In some cases this could be an artefact of the study design: where people are asked retrospectively to indicate a rationale for their actions they can endorse motivations which construct these in a socially desirable light whilst free of other demands. Though it is questionable how far altruism motivates parents to include their children in trials, they may use it functionally as a way of justifying their decision after it has been made. This might be particularly likely where a child has a poor outcome or if a child dies. For bereaved parents, altruism may be a way of finding some small comfort that their child might live on through the lives of those who benefited from the trial [90], and in this way a trial might give some kind of meaning to a loss that otherwise seems unjust and meaningless [89]. It can also be a way of making sense of a complex situation. Entering a child into a trial confers no guaranteed benefit whilst introducing an element of uncertainty into the child's treatment. In reflecting on such a difficult decision parents might look for some positive anchor with which to justify what in some respects is a counterintuitive step. It may be that the promise of benefit to a "future generation" provides such an anchor [90]. It is difficult to uncover these nuances in how families make sense of trials in studies that use questionnaires. The same criticism applies to qualitative research which treats participants' accounts as faithful descriptions of their experiences, rather than as constructions which need to be interpreted in the light of the social context and functions they serve [93,94].

### Sharing responsibility with the child

We have argued that responsibility rather than autonomy is central to understanding the situation of parents when they are approached about trials in paediatrics. However, parents face a new set of issues when their child is old enough to have an opinion on whether they wish to participate in a trial, when the need to protect one's child might come into conflict with the child's wishes. The available evidence indicates that parents negotiate this by allowing the young person to have "their say" in the decision, whilst retaining responsibility for the final decision themselves [95]. Under certain circumstances parents may encourage or persuade children to take part in research: parents of children who had asthma, diabetes, epilepsy or no chronic condition who were considering hypothetical trials [96] and mothers of children with diabetes considering entry to a real trial [84] were prepared to persuade their child to enter a study if they believed that the benefits of participation outweighed the child's fears. Particularly where the child has a life-threatening illness some parents will exclude the child from the decisions about research altogether in order to protect them from distressing information, while other parents will include the child in discussions but make the final decision themselves [97]. This contrasts with studies of hypothetical trials where parents tend to report that the decision-making process was a collaboration with their child. It is likely that the degree of collaboration is related to the threat to the child [7,96,98] and to the cognitive capacity of the child [96]. Considered alongside a study examining willingness to participate in hypothetical asthma trials which suggested that adolescents were significantly more willing than parents to enrol in above-minimal risk research [99], this raises further complexities for paediatric trialists. The parents' move from active participant to passive observer in trial decisions as their child grows older is an important area for future research [7].

## Summary

• In contrast to the autonomy that typifies the situation of adults when they are themselves approached to participate in trials, when parents are asked to consider entering a child in a trial their decision might best be characterised as one that embodies an obligation of responsibility.

• Some parents will see trials as a threat to their child or fear regretting their decision, whilst others see trials as offering hope of better treatments for their child. Almost all parents cherish their role in protecting children and want to secure the best outcome for them, but many are aware of complexities of the medical and research context and how this constrains their fulfilment of this role.

• The conduct of trials with children may benefit from a better understanding of the special situation of parents and their particular need for support that allows them to retain a sense that they have safeguarded their child's interests.

• Research is needed on how this may be realised in practice whilst observing medico-legal requirements. Thinking of consent as a social recognition of the parental role and aiming for 'best possible consent' may open up opportunities to develop approaches to trial communication which incorporate the flexibility for parents to construct what is for them a meaningful understanding of the research, as well as a meaningful exercise of parental responsibility.

## Competing interests

The authors declare that they have no competing interests.

## Authors' contributions

VS and BY contributed equally to the preparation of this manuscript.

## Pre-publication history

The pre-publication history for this paper can be accessed here:


